# Supplementary material for: The role of education, religiosity and development on support for violent practices among Muslims in thirty-five countries
Source: PLoS One. 2021 Nov 24;16(11):e0260429. doi: 10.1371/journal.pone.0260429 (PMC8612526; doi:10.1371/journal.pone.0260429)
Supplement: S1 Appendix — This appendix contains tables of models using alternate specifications of religiosity, education, and development. (PDF) [file pone.0260429.s001.pdf]

Supplementary Material

Table S.1. Results from multilevel logit models predicting support for each component of the violent practices measure among Muslims.

	Death for Apostasy	Stoning Adulterers	Corporal punishment
Intercept	−1.308 (0.239)***	−0.697 (0.190)***	−0.570 (0.166)***
Mosque attendance	0.236 (0.082)**	0.291 (0.066)***	0.344 (0.080)***
Importance of religion	0.355 (0.138)**	0.317 (0.108)**	0.286 (0.092)**
Frequency of prayer	0.440 (0.108)***	0.458 (0.077)***	0.498 (0.110)***
Education quantile	−0.316 (0.080)***	−0.128 (0.060)*	−0.099 (0.059)
Income quantile	−0.018 (0.035)	0.000 (0.031)	−0.055 (0.031)
Age (ref. 18-24)			
Age 25-29	−0.010 (0.048)	0.039 (0.045)	0.005 (0.044)
Age 30-34	0.017 (0.051)	−0.067 (0.048)	−0.016 (0.046)
Age 35-39	−0.131 (0.054)*	−0.068 (0.050)	−0.120 (0.049)*
Age 40-44	−0.159 (0.057)**	−0.193 (0.053)***	−0.143 (0.051)**
Age 45-49	−0.081 (0.061)	−0.115 (0.057)*	−0.067 (0.055)
Age 50-54	−0.163 (0.066)*	−0.161 (0.061)**	−0.109 (0.058)
Age 55-59	−0.230 (0.072)**	−0.164 (0.066)*	−0.147 (0.064)*
Age 60 and over	−0.083 (0.068)	−0.142 (0.062)*	−0.088 (0.060)
Female	−0.027 (0.034)	−0.034 (0.031)	−0.030 (0.031)
Urban	−0.116 (0.033)***	−0.102 (0.030)***	−0.131 (0.029)***
Denomination (ref. Sunni)			
Shia	−0.437 (0.067)***	−0.271 (0.063)***	−0.258 (0.065)***
Other denomination	−0.217 (0.102)*	−0.163 (0.091)	0.014 (0.087)
Just a Muslim	−0.318 (0.041)***	−0.180 (0.039)***	−0.209 (0.036)***
Sufi (ref. non-Sufi)	0.139 (0.041)***	0.099 (0.040)*	0.156 (0.039)***
Necessary to believe in god to be moral	0.071 (0.042)	0.185 (0.040)***	0.095 (0.037)*
Islam is the one true faith	0.399 (0.047)***	0.466 (0.041)***	0.486 (0.040)***
One way to interpret relig. teachings	0.084 (0.033)*	0.065 (0.032)*	−0.002 (0.031)
Religion in conflict with modernity	0.209 (0.038)***	0.212 (0.030)***	0.147 (0.034)***
Dislikes western culture	0.275 (0.034)***	0.347 (0.031)***	0.295 (0.031)***
Western culture is immoral	0.061 (0.036)	0.144 (0.033)***	0.117 (0.032)***
Prefers strong leader to democracy	−0.043 (0.032)	0.064 (0.031)*	0.105 (0.030)***
Socially conservative scale, death	−0.080 (0.039)*	−0.075 (0.036)*	−0.126 (0.034)***
Socially conservative scale, sex	−0.168 (0.037)***	0.051 (0.035)	0.059 (0.033)
Human development index	−0.621 (0.469)	−0.711 (0.405)	−0.637 (0.320)*
HDI x education quantile	0.100 (0.134)	0.096 (0.108)	0.075 (0.083)
N (individual)	33164	33021	33382
N (country)	35	35	35
BIC	29979	33658	35630
SD (education quantile)	0.397	0.285	0.290
SD (attendance)	0.346	0.259	0.360
SD (religion important)	0.705	0.540	0.449

	Death for Apostasy	Stoning Adulterers	Corporal punishment
SD (prayer)	0.495	0.342	0.562
r(intercept, education)	0.700	0.528	0.398
r(intercept, attendance)	−0.662	−0.250	−0.339
r(intercept, religion important)	−0.030	−0.102	−0.037
r(intercept, prayer)	0.334	0.363	0.262
r(education, attendance)	−0.796	−0.575	−0.500
r(education, religion important)	−0.523	−0.434	−0.644
r(education, prayer)	0.485	0.604	0.748
r(attendance, religion important)	0.361	0.365	−0.053
r(attendance, prayer)	−0.587	−0.306	−0.134
r(religion important, prayer)	−0.203	−0.514	−0.390

$^{***}p < 0.001$; $^{**}p < 0.01$; $^{*}p < 0.05$

Notes: All models include random country-level intercepts and slopes for some variables. All quantitative variables are divided by twice their standard deviation for comparability. Results are based on five complete datasets with imputation for missing values.

Table S.2. Results from multilevel models predicting support for violent practices for violating norms among Muslims using GDP to measure to development.

	Model 1	Model 2
Intercept	−0.197 (0.084)*	−0.196 (0.084)*
Mosque attendance	0.112 (0.022)***	0.110 (0.022)***
Importance of religion	0.090 (0.032)**	0.087 (0.032)**
Frequency of prayer	0.157 (0.034)***	0.161 (0.034)***
Education quantile	−0.059 (0.022)**	−0.058 (0.022)**
Income quantile	−0.009 (0.010)	−0.009 (0.010)
Age (ref. 18-24)		
Age 25-29	0.006 (0.014)	0.006 (0.014)
Age 30-34	−0.008 (0.015)	−0.008 (0.015)
Age 35-39	−0.043 (0.016)**	−0.043 (0.016)**
Age 40-44	−0.054 (0.016)***	−0.054 (0.016)**
Age 45-49	−0.033 (0.018)	−0.033 (0.018)
Age 50-54	−0.047 (0.019)*	−0.046 (0.019)*
Age 55-59	−0.061 (0.021)**	−0.060 (0.021)**
Age 60 and over	−0.025 (0.019)	−0.024 (0.019)
Female	−0.011 (0.010)	−0.011 (0.010)
Urban	−0.048 (0.009)***	−0.048 (0.009)***
Denomination (ref. Sunni)		
Shia	−0.130 (0.020)***	−0.129 (0.020)***
Other denomination	−0.049 (0.030)	−0.049 (0.030)
Just a Muslim	−0.086 (0.012)***	−0.086 (0.012)***
Sufi (ref. non-Sufi)	0.065 (0.014)***	0.065 (0.014)***
Necessary to believe in god to be moral	0.035 (0.012)**	0.036 (0.012)**
Islam is the one true faith	0.150 (0.013)***	0.150 (0.013)***
One way to interpret relig. teachings	0.015 (0.010)	0.015 (0.010)

	Model 1	Model 2
Religion in conflict with modernity	$0.077\ (0.010)^{***}$	$0.077\ (0.010)^{***}$
Dislikes western culture	$0.119\ (0.010)^{***}$	$0.119\ (0.010)^{***}$
Western culture is immoral	$0.034\ (0.010)^{***}$	$0.034\ (0.010)^{***}$
Prefers strong leader to democracy	0.018 (0.010)	0.018 (0.010)
Socially conservative scale, death	$-0.034\ (0.011)^{**}$	$-0.034\ (0.011)^{**}$
Socially conservative scale, sex	−0.011 (0.011)	−0.011 (0.011)
GDP per capita (logged)	$-0.292\ (0.129)^{*}$	$-0.276\ (0.131)^{*}$
log GDP x education quantile		0.051 (0.029)
N (individual)	31528	31528
N (country)	35	35
BIC	72174	72187
SD (education quantile)	0.114	0.112
SD (attendance)	0.099	0.100
SD (religion important)	0.162	0.164
SD (prayer)	0.177	0.177
r(intercept, education)	0.368	0.373
r(intercept, attendance)	−0.305	−0.285
r(intercept, religion important)	0.185	0.190
r(intercept, prayer)	0.657	0.642
r(education, attendance)	−0.634	−0.622
r(education, religion important)	−0.481	−0.504
r(education, prayer)	0.743	0.734
r(attendance, religion important)	0.051	0.074
r(attendance, prayer)	−0.362	−0.375
r(religion important, prayer)	−0.213	−0.222

$^{***}p < 0.001$; $^{**}p < 0.01$; $^{*}p < 0.05$

Notes: All models include random country-level intercepts and slopes for some variables. All quantitative variables are divided by twice their standard deviation for comparability. Results are based on five complete datasets with imputation for missing values.

Table S.3. Results from multilevel models predicting support for violent practices for violating norms among Muslims using three category educational variable.

	Model 1	Model 2
Intercept	$-0.207\ (0.084)^{*}$	$-0.203\ (0.084)^{*}$
Mosque attendance	$0.106\ (0.023)^{***}$	$0.105\ (0.023)^{***}$
Importance of religion	$0.086\ (0.032)^{**}$	$0.084\ (0.032)^{**}$
Frequency of prayer	$0.162\ (0.035)^{***}$	$0.164\ (0.035)^{***}$
Secondary	$-0.066\ (0.022)^{**}$	$-0.056\ (0.021)^{**}$
Post-Secondary	$-0.066\ (0.030)^{*}$	−0.057 (0.030)
Income quantile	−0.009 (0.010)	−0.009 (0.010)
Age (ref. 18-24)		
Age 25-29	0.003 (0.014)	0.002 (0.014)
Age 30-34	−0.012 (0.015)	−0.012 (0.015)

	Model 1	Model 2
Age 35-39	-0.048 (0.016)**	-0.048 (0.016)**
Age 40-44	-0.060 (0.016)***	-0.060 (0.016)***
Age 45-49	-0.038 (0.018)*	-0.038 (0.018)*
Age 50-54	-0.055 (0.019)**	-0.055 (0.019)**
Age 55-59	-0.068 (0.021)***	-0.068 (0.021)***
Age 60 and over	-0.028 (0.019)	-0.026 (0.019)
Female	-0.010 (0.010)	-0.010 (0.010)
Urban	-0.050 (0.009)***	-0.050 (0.009)***
Denomination (ref. Sunni)		
Shia	-0.131 (0.020)***	-0.131 (0.020)***
Other denomination	-0.048 (0.030)	-0.048 (0.030)
Just a Muslim	-0.086 (0.012)***	-0.087 (0.012)***
Sufi (ref. non-Sufi)	0.065 (0.014)***	0.066 (0.014)***
Necessary to believe in god to be moral	0.037 (0.012)**	0.037 (0.012)**
Islam is the one true faith	0.150 (0.013)***	0.150 (0.013)***
One way to interpret relig. teachings	0.016 (0.010)	0.016 (0.010)
Religion in conflict with modernity	0.077 (0.010)***	0.077 (0.010)***
Dislikes western culture	0.119 (0.010)***	0.118 (0.010)***
Western culture is immoral	0.034 (0.010)***	0.034 (0.010)***
Prefers strong leader to democracy	0.018 (0.010)	0.017 (0.010)
Socially conservative scale, death	-0.033 (0.011)**	-0.033 (0.011)**
Socially conservative scale, sex	-0.012 (0.011)	-0.012 (0.011)
Human development index	-0.335 (0.133)*	-0.260 (0.136)
HDI x Secondary		0.111 (0.039)**
HDI x Post-Secondary		0.076 (0.043)
N (individual)	31528	31528
N (country)	35	35
BIC	72229	72252
SD (Secondary)	0.108	0.099
SD (Post-Secondary)	0.151	0.149
SD (attendance)	0.106	0.105
SD (religion important)	0.162	0.162
SD (prayer)	0.183	0.183
r(intercept, secondary)	0.280	0.267
r(intercept, post-secondary)	0.306	0.283
r(intercept, attendance)	-0.219	-0.212
r(intercept, religion important)	0.183	0.184
r(intercept, prayer)	0.598	0.590
r(secondary, attendance)	-0.297	-0.341
r(post-secondary, attendance)	-0.698	-0.720
r(secondary, religion important)	-0.330	-0.394
r(post-secondary, religion important)	-0.487	-0.514
r(secondary, prayer)	0.326	0.338
r(post-secondary, prayer)	0.754	0.762
r(secondary, post-secondary)	0.754	0.750
r(attendance, religion important)	0.196	0.206
r(attendance, prayer)	-0.467	-0.471

	Model 1	Model 2
r(religion important, prayer)	−0.263	−0.266

$^{***}p < 0.001$; $^{**}p < 0.01$; $^{*}p < 0.05$

Notes: All models include random country-level intercepts and slopes for some variables. All quantitative variables are divided by twice their standard deviation for comparability. Results are based on five complete datasets with imputation for missing values.
